# Meta‐Analysis of Consumer Willingness to Pay for Short Food Supply Chain Products

**DOI:** 10.1002/gch2.202400154

**Published:** 2025-01-27

**Authors:** Muhammad Adzran Che Mustapa, Zein Kallas

**Affiliations:** ^1^ Centre for Agrofood Economics and Development‐UPC‐IRTA (CREDA) Universitat Politècnica de Catalunya (UPC) Castelldefels 08860 Spain

**Keywords:** meta‐analysis, meta‐regression, short food supply chain, willingness to pay

## Abstract

Due to their perceived benefits for health, the environment, the economy, and sustainability, in recent years there has been a growing interest on the part of researchers and policymakers in short‐food supply chains (SFSCs). However, a systematic review of the literature on this topic remains lacking. To address this gap, the study conducts a meta‐analysis to examine consumer willingness to pay (WTP) for SFSC products, taking into account various sociodemographic factors and sustainability attributes. On average, consumers are willing to pay a 34.5% premium for SFSC products. Key factors influencing WTP include gender, education, study year, age, region, product category, and sustainability attributes. Women exhibit higher WTP, and individuals with higher education levels also demonstrate increased WTP. Notably, WTP for SFSC products is lower before 2014, while younger consumers exhibit higher WTP overall. Consumers in Western Europe present higher WTP estimates compared to those in North America and other regions. In terms of sustainability attributes, organic products receive the highest WTP, with food categories such as meat, poultry, dairy products, and honey showing the highest estimates. These findings offer valuable insights for SFSC producers, marketers, and policymakers, guiding effective strategies to promote SFSC products within sustainable agri‐food systems.

## Graphical abstract

This study conducts a meta‐analysis of consumer willingness to pay (WTP) for short food supply chain (SFSC) products, addressing a gap in the literature. The findings reveal an average 34.5% premium for SFSC products, with WTP influenced by factors such as gender, education, age, region, and sustainability attributes. Women, younger consumers, and those with higher education levels exhibit higher WTP, with Western Europe showing the highest regional estimates. Organic products and categories like meat, poultry, dairy, and honey receive the highest WTP, offering actionable insights for SFSC stakeholders to promote sustainable agri‐food systems.

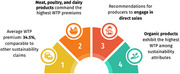

## 1 Introduction

### 1.1 Research Background

The global food system is increasingly shifting toward sustainability, local sourcing, and direct producer‐consumer relationships.^[^ ^1^ ^]^ Short food supply chains (SFSCs) have gained prominence due to growing consumer interest in the environmental and health aspects of food systems.^[^ ^2^ ^]^ These systems enhance sustainability in the agri‐food market by offering high‐quality products, reducing post‐harvest losses, and minimizing reliance on intermediaries.^[^ ^3^, ^4^ ^]^ SFSCs also promote food security in low‐income regions and meet consumer demand for fresher, locally sourced products in high‐income areas.^[^ ^1^ ^]^ Additionally, they reduce the environmental impact of long‐distance food transport, preserve local culinary traditions, foster social cohesion, and encourage community engagement through activities such as farmers’ markets.^[^ ^5^, ^6^ ^]^

SFSCs can be categorized by type into direct, short‐distance, and extended. Direct chains involve farmers selling directly to consumers, short‐distance chains focus on local sales near production sites, and extended chains enable wider distribution through specialized stores.^[^ ^7^, ^8^ ^]^ Regulation (EU) No 1305/2013 defines SFSCs as involving a limited number of economic operators working together to maintain close geographical and social relationships while promoting local economic development.^[^ ^9^, ^10^ ^]^

What sets SFSCs apart is their emphasis on reducing intermediaries and ensuring fair prices for producers, while integrating social and economic sustainability principles such as fair trade.^[^ ^11^, ^12^ ^]^ Unlike local food movements, which primarily emphasize geographical proximity, SFSCs focus on fostering direct relationships^[^ ^13^, ^14^, ^15^, ^16^, ^17^ ^]^ that strengthen trust and knowledge exchange between producers and consumers,^[^ ^18^ ^]^ while reducing environmental impact.^[^ ^13^, ^19^, ^20^ ^]^ Innovations in SFSCs, such as adopting electric vehicles (EVs), have further reduced greenhouse gas emissions and transportation costs.^[^ ^21^ ^]^ Additionally, social media platforms are increasingly vital with regard to SFSCs, strengthening connections from farm to fork.^[^ ^22^ ^]^ In this context, consumers are increasingly showing positive attitudes and willingness to pay (WTP) premium prices for SFSC products. These preferences are shaped by various factors, particularly the effective communication of their environmental and social benefits, and consumer understanding of their sustainable implications.

### 1.2 Research Gap

Existing meta‐analyses with regard to consumer WTP have primarily focused on specific sustainability attributes such as sustainable food products,^[^ ^23^ ^]^ local foods,^[^ ^24^ ^]^ and farm animal welfare.^[^ ^25^ ^]^ Recently, Jia et al.^[^ ^1^ ^]^ conducted a systematic review of the literature on SFSCs, exploring factors influencing their adoption, sustainable practices, challenges, stakeholders, and impacts. This review highlighted key drivers, including the increasing demand for local food, consumer preferences for sustainability, and the role of transparency in promoting SFSC adoption.

Although Chiaverina et al.^[^ ^26^ ^]^ analyzed the economic performance of farms engaged in SFSCs through a meta‐analysis, no previous study has systematically examined consumers' WTP for SFSC products. Addressing this gap, the current study provides a comprehensive meta‐analysis synthesizing insights from the existing literature on consumer WTP for SFSC products. By doing so, it offers a deeper understanding of the factors influencing consumer behavior and supports the development of more effective strategies to promote SFSCs.

### 1.3 Aim of the Study and Contributions

The main objectives of this study are to: 1) establish the range of WTP for SFSC products compared to traditional supply chains, 2) assess whether the WTP range aligns with findings related to other sustainability claims, 3) identify food categories with the highest relative premium for SFSC, and 4) analyze the factors contributing to variability in WTP.

This study provides actionable insights for decision‐makers, governments, and sustainable food producers to formulate effective policies that support sustainable development. By shedding light on consumers’ WTP for SFSC products, it highlights strategies to enhance the economic viability of SFSCs, strengthen local economies, and increase the availability of high‐quality, fresh products. Additionally, it deepens the understanding of consumer preferences, offering a foundation for innovative diversification strategies within the agri‐food system. Beyond these immediate contributions, the study adds to the broader discourse on sustainability by providing recommendations for future research and practice. It underscores the role of SFSCs in fostering economic resilience, sustainability, and consumer trust in modern food systems.

## 2 Materials and Methods

Meta‐analysis is a statistical method that combines results from multiple independent studies to provide a single, clearer estimate of the overall effect, increasing both the accuracy and reliability of the findings.^[^ ^27^ ^]^ This method extends beyond qualitative literature reviews by providing a quantitative synthesis of the data which allows for a more objective appraisal of evidence.^[^ ^28^ ^]^ Scholars from various fields including psychology, education, marketing, and the social sciences often use meta‐analyses to synthesize the findings of previous studies and to control the effects of specific characteristics (for specific products or applied methods) with regard to WTP estimates.^[^ ^29^ ^]^

### 2.1 Literature Search and Selection Criteria

**Figure 1:**
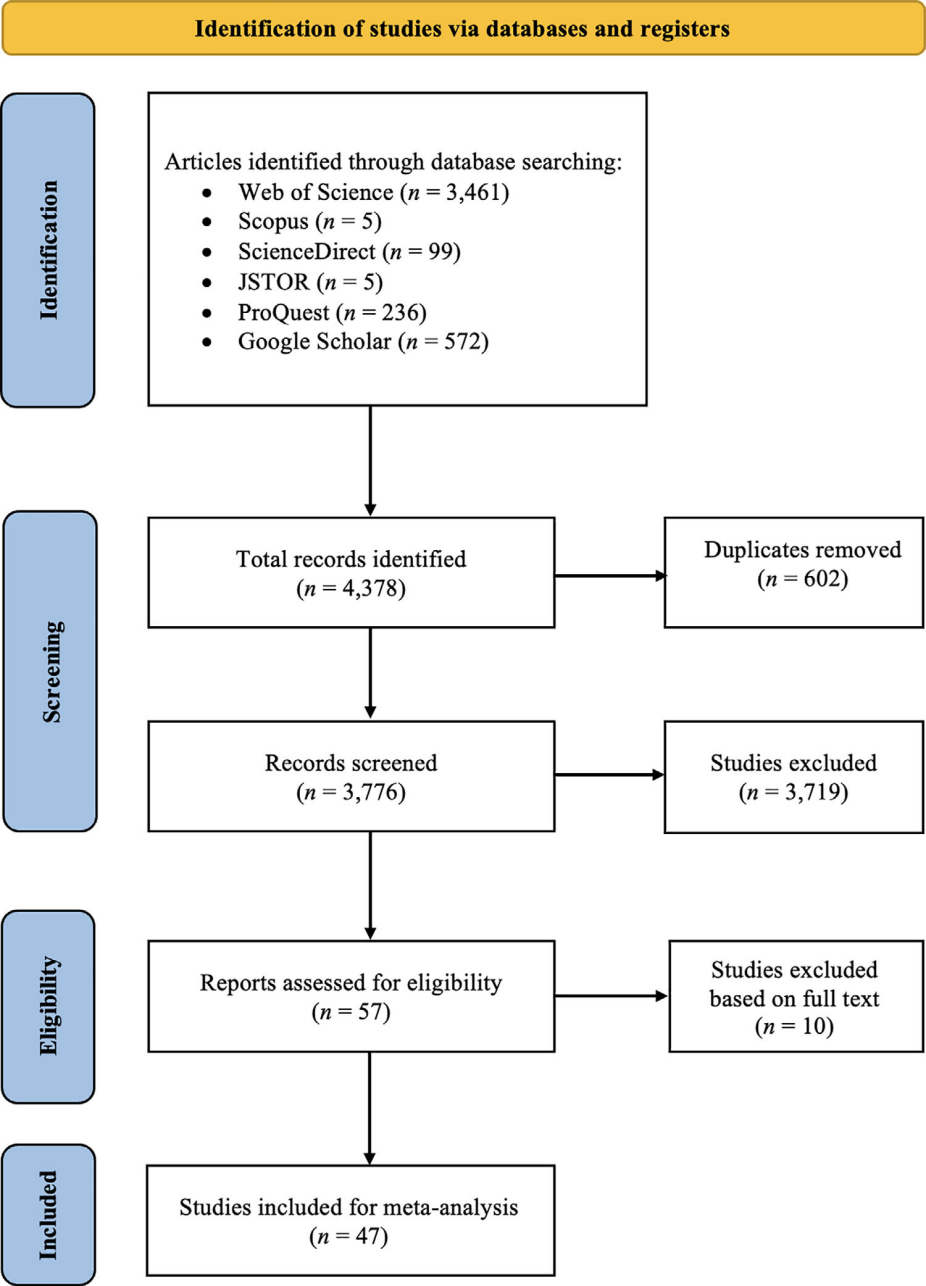
Flow diagram of the search and selection process based on the Preferred Reporting Items for Systematic Reviews and Meta‐Analyses (PRISMA) template.

The study followed the PRISMA (Preferred Reporting Items for Systematic Reviews and Meta‐Analysis) guidelines^[^ ^30^ ^]^ (**Figure** **1**), which ensure transparency and rigor in the review process. The review protocol, which contains information about the search terms, databases, eligibility criteria, and selection process, is presented below. For this study, to ensure that a transparent approach was maintained during the literature search for the meta‐regression analysis (MRA), we adhered to the guidelines outlined in ‘Meta‐Analysis of Economics Research Reporting Guidelines’ as presented by Stanley et al.^[^ ^29^ ^]^

The systematic literature search and the data extraction process were conducted by two independent reviewers (Author A and Author B), both of whom are experienced in the field and not affiliated with the studies under review. The data extraction process involved a thorough review of each selected study to ensure the accurate and consistent extraction of relevant data. Any discrepancies or conflicts between reviewers regarding the inclusion or exclusion of a particular paper were resolved through discussion and consensus. In instances where consensus could not be achieved, an external researcher from the CREDA research center was consulted to make the final decision, ensuring an unbiased resolution.

In this study, we used the following electronic databases: i) Web of Science (WOS) (https://www.webofscience.com/), Scopus (https://www.scopus.com/), ScienceDirect (https://www.sciencedirect.com/), Journal STOrage (JSTOR) (https://www.jstor.org/), ProQuest (https://www.proquest.com/), and Google Scholar (https://scholar.google.com/). The search, spanning 20 years, included articles published in English between 2003 and 2023.

To improve the accuracy of the search results, we used Boolean strings, which involve combining keywords with terms such as ‘AND’ and ‘OR’ to refine the search. We examined titles, abstracts, and article keywords. The original studies included in this meta‐analysis were identified through keyword searches in the relevant literature databases. We used the keywords ‘consumer*’, ‘willingness to pay’, ‘WTP’, ‘short food supply chains’, and ‘short supply chains’ to ensure that the relevant literature was identified.

### 2.2 Eligibility Criteria

All the articles that were included in our analysis of consumer WTP for SFSC products were published in English in peer‐reviewed journals between January 2003 and September 2023. Notably, literature reviews, theses and dissertations, letters, book chapters, reports, author comments, and other grey literature were not included. We focused on peer‐reviewed academic articles to ensure the inclusion of high‐quality and reliable data. By prioritizing such sources, we aimed to minimize the risk of bias and enhance the robustness of our review process. Additionally, peer‐reviewed publications undergo rigorous scrutiny by experts in the field, providing greater confidence in the accuracy and validity of the information presented.

### 2.3 Process of Study Selection

Figure 1 portrays the results of the search conducted using the PRISMA template. PRISMA is a widely used tool that helps ensure systematic and clear reporting of search results in reviews.^[^ ^31^ ^]^ The search yielded 4,378 published papers, including 602 duplicates and papers obtained using a range of different search engines. After eliminating the duplicates and papers that did not meet the inclusion criteria, we were left with 3,776 articles; of these, 3,719 were deemed unsuitable for our analysis. For example, 57 articles did not include measures of WTP or dealt only with short supply chains.

To minimize the potential impact of publication bias on our meta‐analysis, we implemented a comprehensive strategy to search unpublished literature.^[^ ^32^, ^33^ ^]^ This involved grey literature searches, contacting researchers, and considering preprint archives. By taking these steps, we aimed to minimize the risk of excluding valuable, unpublished research that might have influenced the outcomes of our review. This approach allowed us to access a broader range of evidence beyond peer‐reviewed journal articles, thereby enhancing the comprehensiveness and validity of our findings.

As uniform interpretation of the measured quantity is crucial for the reliability of MRA,^[^ ^34^, ^35^ ^]^ we evaluated the 57 remaining articles. Among them, we identified 47 articles that included comparable measure(s) of WTP with clearly reported units (such as USD or EUR). The remaining 10 studies did not meet the criteria for uniform interpretation for various reasons. **Table** **1** provides an overview of the 47 articles used for the MRA in chronological order, presenting the year of publication, authors, journals, number of participants, origin, and type of articles analyzed, along with the WTP estimates reported in each article.

**Table 1:** Characteristics of the studies considered in the analysis.

No.	Study	Year	Country	Number of samples	Food products	Method	Average yearly income (USD)	Proportion of post‐graduate studies	Average age	Female
1	Adalja et al.^[^ ^36^ ^]^	2015	USA	358	Beef	Non‐hypothetical	29,565.82	0.895	42.70	0.851
2	Adalja et al.^[^ ^36^ ^]^	2015	USA	327	Beef	Non‐hypothetical	30,657.31	0.828	47.30	0.585
3	Alfnes and Sharma^[^ ^37^ ^]^	2010	USA	322	Beef, chicken, and pork	Hypothetical	29,776.04	0.470	45.50	0.800
4	Ay et al.^[^ ^38^ ^]^	2017	France	111	Organic wines	Non‐hypothetical	33,127.62	0.540	43.05	0.516
5	Ay et al.^[^ ^38^ ^]^	2017	France	111	Non‐organic wines	Non‐hypothetical	33,127.62	0.540	43.05	0.516
6	Berg and Preston^[^ ^39^ ^]^	2017	New Zealand	137	Oranges, tomatoes, garlic	Non‐hypothetical	37,798.09	0.610	39.80	0.630
7	Boys et al.^[^ ^40^ ^]^	2014	Dominica	188	Fruits and vegetables	CVM (hypothetical)	29,444.92	0.17	36.85	0.543
8	Carpio and Isengildina‐Massa^[^ ^41^ ^]^	2009	USA	500	Local products	CVM (hypothetical)	17,678.47	0.92	52.51	0.515
9	Carpio and Isengildina‐Massa^[^ ^41^ ^]^	2009	USA	500	Local animal products	CVM (hypothetical)	17,678.47	0.92	52.51	0.515
10	Chang et al.^[^ ^42^ ^]^	2013	USA	212	Rib‐eye steak (Beef)	Non‐hypothetical	24,378.74	0.514	50.52	0.703
11	Darby et al.^[^ ^43^ ^]^	2008	USA	267	Strawberries	Non‐hypothetical	75,767.86	0.789	49.20	0.694
12	Darby et al.^[^ ^43^ ^]^	2008	USA	263	Strawberries	Non‐hypothetical	87,962.92	0.773	49.80	0.747
13	de‐Magistris and Gracia^[^ ^44^ ^]^	2016	Spain	171	Local almonds	Non‐hypothetical CE	31,118.98	0.373	46.56	0.519
14	de‐Magistris and Gracia^[^ ^44^ ^]^	2016	Spain	171	Organic almonds	Non‐hypothetical CE	31,118.98	0.373	46.56	0.519
15	Feucht and Zander,^[^ ^45^ ^]^	2017	Germany	1001	UHT Milk	CE (hypothetical)	46,974.00	0.237	43.31	0.497
16	Feucht and Zander^[^ ^45^ ^]^	2017	Spain	1002	UHT Milk	CE (hypothetical)	31,630.87	0.442	43.41	0.501
17	Feucht and Zander^[^ ^45^ ^]^	2017	France	1000	UHT Milk	CE (hypothetical)	42,789.66	0.469	42.78	0.508
18	Feucht and Zander^[^ ^45^ ^]^	2017	Italy	1003	UHT Milk	CE (hypothetical)	33,050.19	0.343	43.29	0.506
19	Feucht and Zander^[^ ^45^ ^]^	2017	Norway	1001	UHT Milk	CE (hypothetical)	48,783.86	0.580	43.29	0.514
20	Feucht and Zander,^[^ ^45^ ^]^	2017	The UK	1000	UHT Milk	CE (hypothetical)	37,036.16	0.522	42.12	0.501
21	Galati et al.^[^ ^10^ ^]^	2022	Italy	273	Oranges	Hypothetical	22,136.12	0.630	39.00	0.690
22	Gracia et al.^[^ ^46^ ^]^	2014	Spain	803	Organic eggs	CE (hypothetical)	29,201.05	0.363	45.43	0.545
23	Gracia et al.^[^ ^46^ ^]^	2014	Spain	803	Free‐range eggs	CE (hypothetical)	29,201.05	0.363	45.43	0.545
24	Grebitus et al.^[^ ^47^ ^]^	2013	Germany	47	Apples	Non‐hypothetical	34,250.16	0.590	43.00	0.640
25	Grebitus et al.^[^ ^47^ ^]^	2013	Germany	47	Wines	Non‐hypothetical	34,250.16	0.590	43.00	0.640
26	Hempel and Hamm^[^ ^48^ ^]^	2016	Germany	159	Butter	CE (hypothetical)	41,410.55	0.340	45.65	0.591
27	Hempel and Hamm^[^ ^48^ ^]^	2016	Germany	159	Butter	CE (hypothetical)	26,857.12	0.308	47.44	0.648
28	Hempel and Hamm^[^ ^48^ ^]^	2016	Germany	155	Butter	CE (hypothetical)	27,728.24	0.271	42.35	0.665
29	Hempel and Hamm^[^ ^48^ ^]^	2016	Germany	158	Butter	CE (hypothetical)	36,205.09	0.348	42.37	0.722
30	Kallas et al.^[^ ^49^ ^]^	2019	Argentina	210	Honey	CE (hypothetical)	41,299.51	0.629	41.43	0.708
31	Kiss et al.^[^ ^50^ ^]^	2020	Hungary	1034	Honey, eggs, fruits, and vegetables	Non‐hypothetical	9,974.64	0.500	37.76	0.690
32	Kokthi et al.^[^ ^51^ ^]^	2021	Albania	434	Fruits and vegetables	CVM (hypothetical)	5,133.00	0.560	39.50	0.600
33	Linnes et al.^[^ ^52^ ^]^	2022	USA	454	Local food (fish, shrimp, and coffee)	(Non‐hypothetical)	67,631.77	0.611	41.37	0.594
34	Maples et al.^[^ ^53^ ^]^	2018	USA	297	Tomatoes	CE (hypothetical)	65,560.80	0.579	50.50	0.572
35	Maples et al.^[^ ^53^ ^]^	2018	USA	1,046	Tomatoes	CE (hypothetical)	61,635.08	0.509	48.90	0.531
36	Maples et al.^[^ ^53^ ^]^	2018	USA	1,315	Tomatoes	CE (hypothetical)	74,565.71	0.594	48.40	0.557
37	Maples et al.^[^ ^53^ ^]^	2018	USA	297	Tomatoes	CE (hypothetical)	67,276.28	0.542	47.10	0.522
38	Maples et al.^[^ ^53^ ^]^	2018	USA	293	Tomatoes	CE (hypothetical)	61,035.12	0.523	47.70	0.526
39	Maples et al.^[^ ^53^ ^]^	2018	USA	1,401	Tomatoes	CE (hypothetical)	74,089.65	0.533	46.30	0.473
40	Mazzocchi et al.^[^ ^54^ ^]^	2022	Italy	319	Parma ham	CE (hypothetical)	26,245.80	0.33	32.00	0.550
41	Mazzocchi et al.^[^ ^54^ ^]^	2022	Italy	319	Parma ham	CE (hypothetical)	26,245.80	0.33	32.00	0.550
42	Meas et al.^[^ ^55^ ^]^	2015	USA	912	Blackberry Jam	Non‐hypothetical	56,954.22	0.272	56.00	0.574
43	Meas et al.^[^ ^55^ ^]^	2015	USA	971	Blackberry Jam	Non‐hypothetical	65,300.65	0.335	57.98	0.546
44	Thilmany et al.^[^ ^56^ ^]^	2008	USA	1,549	Melons	(Non‐hypothetical)	32,389.86	0.323	51.07	0.740
45	Wägeli et al.^[^ ^57^ ^]^	2016	Germany	597	Eggs	CE (hypothetical)	27,609.16	0.546	49.42	0.620
46	Wägeli et al.^[^ ^57^ ^]^	2016	Germany	597	Milk	CE (hypothetical)	27,609.16	0.546	49.42	0.620
47	Wägeli et al.^[^ ^57^ ^]^	2016	Germany	597	Pork	CE (hypothetical)	27,609.16	0.546	49.42	0.620

Abbreviations: Choice experiment (CE); contingent valuation method (CVM); United States of America (USA); United Kingdom (UK); milk that has been processed at ultra‐high temperatures (UHT milk).

### 2.4 Data Extraction and Critical Analysis

The chosen effect size was the standardized average WTP value (based on the studies considered in our analysis). This perspective aligns with the approach adopted by Xia and Zeng.^[^ ^58^ ^]^ The ‘mean WTP’ represents the average amount consumers are willing to pay, which reflects their perceptions of value. This allows us to compare how much more people are willing to spend on sustainable products compared with conventional ones.^[^ ^59^ ^]^ WTP, used as the main variable in our analysis, refers to the extra amount (in percentage) consumers are willing to pay over the conventional product price.^[^ ^25^ ^]^ In this study, all the WTP values are presented as percentages.

(1) $$\mathrm{WTP}\;\left(\%\right)=\frac{WTP\;sustainable-P\;conventional}{P\;conventional\;}\;\times 100\%$$

where ‘P conventional’ refers to the price of conventional products.^[^ ^60^ ^]^ We determined the price of such products by searching for the value of such products based on the year of data collection. This approach was adopted in cases where some of the papers included in the review did not mention the price of conventional food products directly. This method ensured that the conventional product prices used in the analysis were reflective of the time period in which the data were collected, allowing for a relatively accurate comparison between sustainable and conventional food product pricing. We also identified key factors, entitled moderator variables, to help explain differences in the data across studies. The variables included the average values of income, age, percentage of the population with more than a university education, and categorical moderators (such as product category, region of study, and study method). Income is expressed as the annual household income (in USD).

### 2.5 Data Analysis

The data were examined and analyzed using Review Manager 5.4 (RevMan 5.4) and Stata 18.0 software. A random‐effects model was applied because the studies analyzed showed high heterogeneity (I^2^ > 50%). This model is particularly suited for addressing such heterogeneity, as it accounts for differences between studies and provides a more realistic average effect size.^[^ ^61^, ^62^ ^]^ Unlike a fixed‐effects model, which assumes all studies share the same underlying effect, the random‐effects model allows for variations across studies. Producing wider confidence intervals reduces the potential bias from smaller studies and minimizes the risk of overestimating the significance of results.^[^ ^62^, ^63^ ^]^ This makes it a recommended approach in meta‐analysis where heterogeneity is expected.^[^ ^64^ ^]^

To ensure the robustness and reliability of the findings, additional statistical analyses were conducted. Egger's test and funnel plots were used to determine whether or not smaller studies had a disproportionate impact on the results. These methods help to identify publication bias, which occurs when studies with significant results are more likely to be published compared to those with non‐significant results.^[^ ^65^, ^66^ ^]^ Additionally, a leave‐one‐out sensitivity analysis was performed. This method systematically removes one study at a time to evaluate its impact on the overall results, confirming that no single study disproportionately influenced the findings.

A subgroup analysis was also conducted to understand the reasons for variability between studies. The data were divided into seven categories based on factors such as age, income, publication year, study region, product category, and method type. The I^2^ statistic was used to measure the proportion of variability between studies due to heterogeneity. Higher I^2^ values indicate greater differences between studies.^[^ ^59^ ^]^ This subgroup analysis provided valuable insights into the factors driving differences in consumer WTP for SFSC products, offering a deeper understanding of consumer behavior.

## 3 Results

### 3.1 Descriptive Statistics

**Figure 2:**
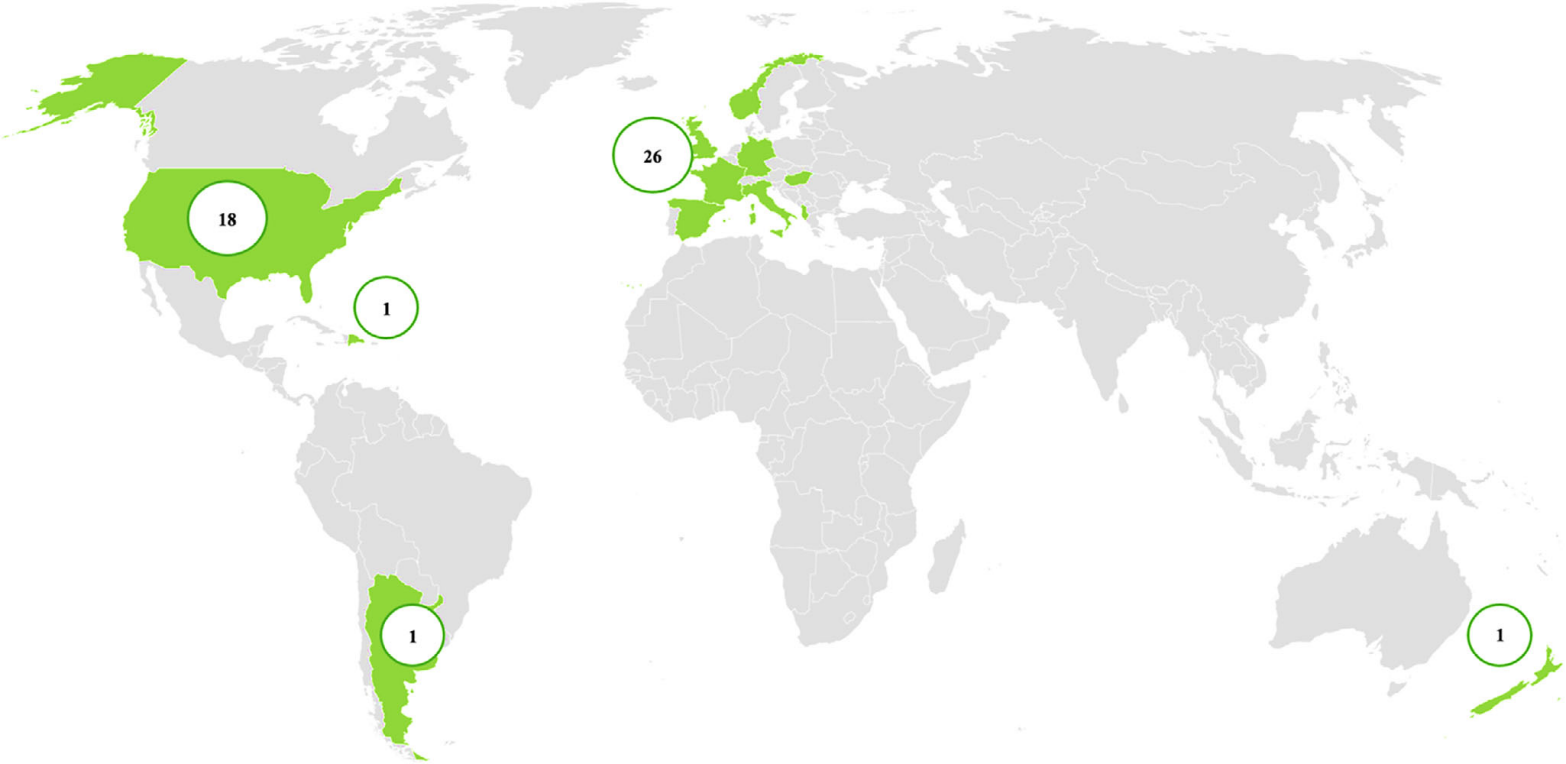
A number of studies on the willingness to pay (WTP) for food in short‐supply chains.

Among the 47 studies considered in this study, 26 were conducted in Europe, 18 in the USA, and one each in Dominica, Argentina, and New Zealand (**Figure** **2**). In this study, we measured diverse WTP estimates for food from short supply chains and their attributes; the sample sizes of individual studies varied, ranging from 1,549 (for the USA) to 111 (for France). Adalja et al.^[^ ^36^ ^]^ reported the highest mean percentage of WTP (62.1%), whereas Gracia et al.^[^ ^46^ ^]^ reported the lowest percentage (2.3%) for local and organic claim products in Spain. The details of the studies are summarized in Table 1.

### 3.2 Overall Results

**Figure 3:**
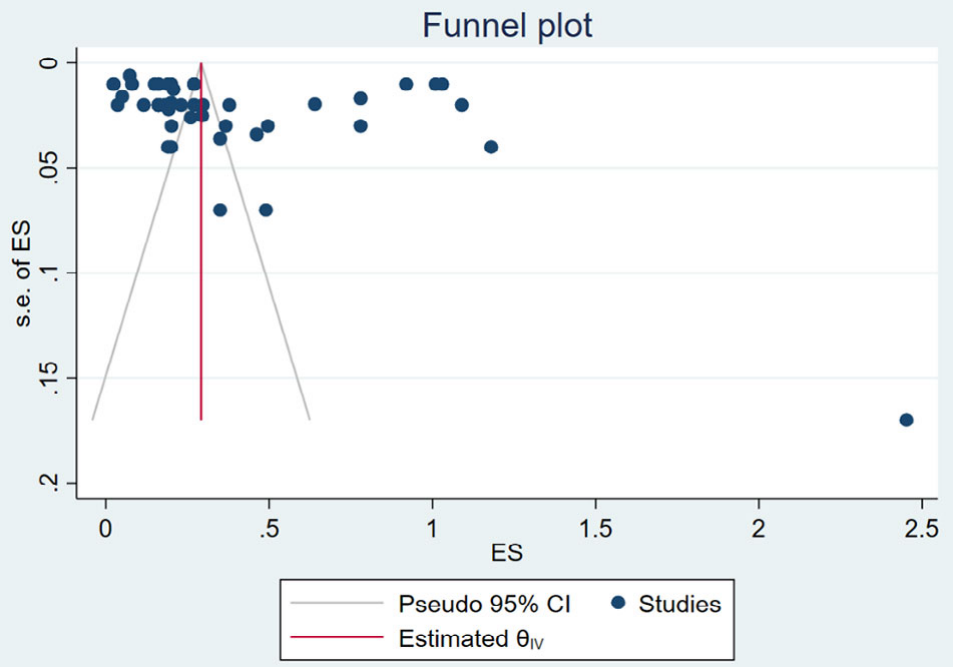
Funnel plot of the study. Note: effects size (ES); confidence interval (CI), willingness to pay (WTP), standard error (s.e.). Note: ES is effect size (standardized average WTP value in this study), and the s.e. of ES denotes the standard error of the effect size. The plot resembles a symmetrical funnel plot with regard to the absence of publication selection bias. The funnel was not symmetrical, indicating a degree of publication bias.

The quality of all the papers considered in this study was high, as shown in the quality assessment graph in **Figure** **3**, which portrays the risk of bias with regard to each study. Additionally, we used Egger's test to evaluate the potential for publication bias in the meta‐analysis through the use of funnel plot asymmetry (**Table** **2**). The Egger's test results confirmed the existence of publication bias in favor of the studies with positive WTP values forSFSCs (*p*<0.01). This asymmetry may be due to the empirical effects noted in the literature.^[^ ^59^ ^]^

**Table 2:** Results of Egger's test (n = 47).

Std_Eff	Coefficient	Standard error	Z	P > |z|	Lower 95 % CI	Upper 95% CI
Slope	0.122	0.067	1.83	0.067	− 0.087	0.252
Bias	10.673	1.995	5.35	0.000^**)^	6.762	14.583

** Significance level: 0.01. *p* = 0.00<0.01, indicating a significant difference, which means the significant existence of publication bias

Abbreviations: Standardized effects (Std_Eff); Z‐score (Z); P‐value for the Z‐test (P > |z|); confidence interval (CI).g.

### 3.3 Sub‐Group Results

**Table** **3** summarizes the sub‐group analyses of the WTP estimates, revealing an overall estimate of 34.5%. With respect to sociodemographic characteristics, older individuals demonstrated a higher WTP (43%) than their younger counterparts (22%), with the lower‐income groups showing a stronger inclination to pay for SFSCs (42%) than the higher‐income groups (23%). When categorized by publication date, articles published after 2014 had a higher WTP estimate (38%) than those published before 2014 (31%). Western Europe had the highest WTP estimate (58%), followed by North America (NA) (33%). In terms of food categories, meat and dairy products portrayed the highest WTP values (54% and 53%, respectively), whereas fruits and vegetables (23%) and beverages (23%) showed the lowest WTP values. The hypothetical and non‐hypothetical methods yielded WTP estimates of 39% and 36%, respectively. The sub‐group analyses provide valuable insights into the factors that shape the willingness of the population considered in this study to pay for SFSC products.

**Table 3:** Summary of the results of the sub‐group analysis.

Sub‐group	WTP estimate (%)	Lower 95% CI (%)	Upper 95% CI (%)	No. of studies	I^2^ (%)
Sub‐group by age					
18–32 years	22	19	26	2	88
33–47 years	29	28	29	33	100
48 and older	43	42	44	12	100
Sub‐group by annual income (USD)					
Less than 30,000	42	22	63	17	100
30,001–60,000	40	27	54	20	100
More than 60,001	23	17	28	10	97
Sub‐group of date by publication					
Before 2014	31	16	46	6	98
After 2014	38	28	49	41	100
Sub‐group by region					
North America	33	24	42	19	99
Western Europe	58	38	78	14	100
Eastern Europe	21	18	23	1	−
Northern Europe	27	25	29	1	−
Southern Europe	18	11	25	10	98
Other regions	25	17	67	2	99
Sub‐group by product category					
Fruits and vegetables	23	19	28	17	96
Meat poultry	54	26	81	10	100
Dairy products	53	31	76	11	100
Eggs	28	7	62	3	100
Honey and other bee products	46	40	52	1	100
Value added products	34	26	42	2	88
Beverages	23	15	31	3	53
Sub‐group by sustainable attribute					
Environmentally friendly	20	17	22	16	93
Local	38	25	50	15	100
Organic	53	30	76	16	100
Sub‐group by method type					
Hypothetical	39	27	51	17	99
Non‐hypothetical	36	24	48	30	100
Overall estimate	34.5	22.7	48.7	47	93.6

WTP estimates indicate the premium in percentage that consumers are willing to pay for SFSC products. I^2^ is the variation in effect size (ES) attributable to heterogeneity; all values were greater than 80%, indicating a high degree of heterogeneity. There were six sub‐groups based on age, annual income, date of publication, region, product category, and method types. Outliers were excluded because of their limited number. Sub‐group analysis was conducted using the RevMan software. Abbreviations: Willingness to pay (WTP); confidence interval (CI).

**Figure 4:**
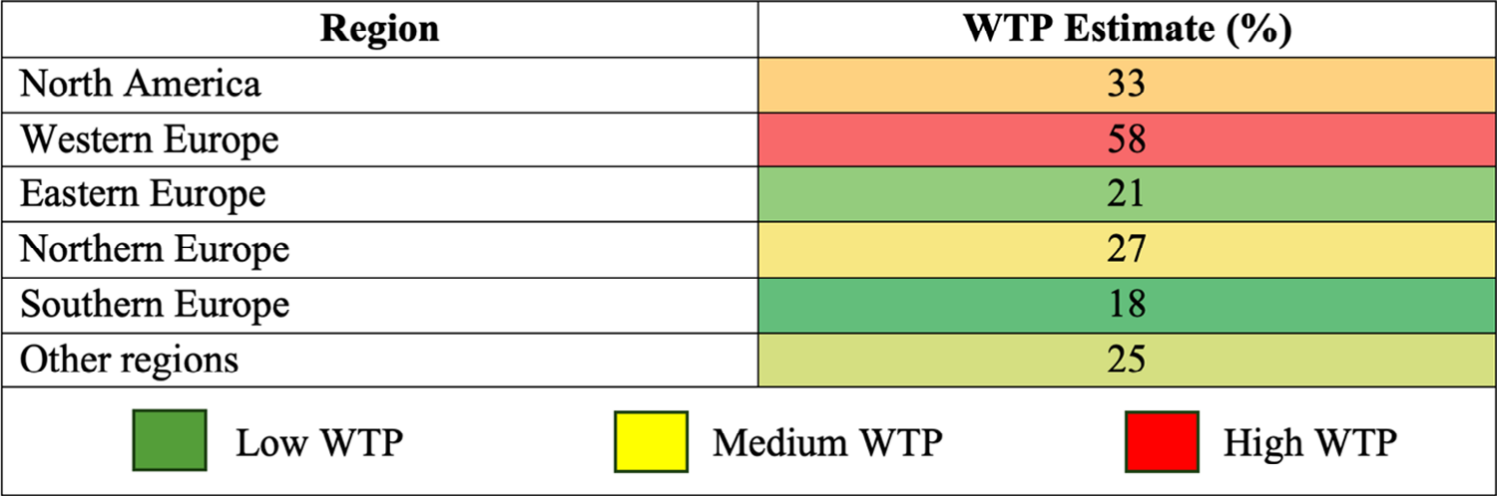
Color‐coded table: regional comparison of average WTP estimates.

**Figure** **4** provides a color‐coded comparison of average WTP estimates across different regions. The results indicate that Western Europe has the highest WTP estimate (58%), suggesting a stronger consumer preference for SFSC products compared to other regions. Conversely, Southern Europe and Eastern Europe exhibit the lowest WTP estimates (18% and 21%, respectively).

**Figure 5:**
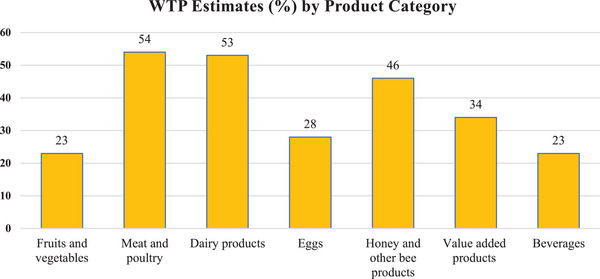
Bar chart: WTP estimates (%) by product category.

**Figure** **5** provides a bar chart comparison of average WTP estimates across different product categories. The results indicate that Meat and Poultry products exhibit the highest WTP estimates (54%), followed closely by Dairy products (53%) and Honey and other bee products (46%). Conversely, Fruits and Vegetables, along with Beverages, show the lowest WTP estimates (23%). These findings suggest that consumers are willing to pay higher premiums for protein‐rich and dairy‐based products, whereas fresh produce and beverages command lower premiums.

**Figure 6:**
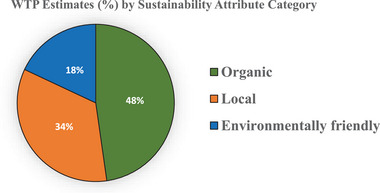
Pie Chart: WTP estimates (%) by sustainability attribute category.

**Figure** **6** provides a pie chart comparison of average WTP estimates across different sustainability attributes. The results show that “Organic” products command the highest WTP estimates (48%), followed by “Local” products (34%). In contrast, “Environmentally Friendly” products exhibit the lowest WTP estimates (18%). These findings indicate a stronger consumer preference for organic and locally sourced products compared to those primarily marketed as environmentally friendly.

### 3.4 MRA Results

We conducted an MRA to determine the source of the heterogeneity in the WTP estimates. The findings are summarized in **Table** **4**. The joint test yielded a *p*‐value of 0.000, indicating that the overall model fit was statistically significant. The I^2^ value was 99.53%, indicating residual variation due to heterogeneity. The R^2^ value was 66.22%, indicating that other sources of heterogeneity should be further investigated and considered in the interpretation of the MRA results. The MRA results indicated that the percentage of females in the population, education, year of publication, age, region, product categories, and sustainability attributes were the main causes of high heterogeneity in this study; these factors significantly affected the variations in the WTP estimates in previous studies.

**Table 4:** Results of meta‐regression analysis (MRA) (excluding outliers).

	Coefficient	Standard error	*p* > |z|
Sociodemographic variables			
Female	1.361	0.644	0.035^*^
Education	0.852	0.318	0.007^**^
Before 2014	−0.521	0.193	0.007^**^
18–32 years old	0.835	0.334	0.013^*^
33–47 years old	0.012	0.128	0.923
<USD30,000	−0.181	0.185	0.329
USD 30,001–60,000	0.222	0.154	0.150
Regions			
North America	1.441	0.465	0.002^**^
Western Europe	1.359	0.440	0.002^**^
Southern Europe	1.196	0.511	0.019^*^
Northern Europe	1.057	0.502	0.035^*^
Product categories			
Fresh produce (fruits and vegetables)	0.499	0.236	0.035^*^
Meat and poultry	0.590	0.257	0.022^*^
Dairy products (fresh milk and cheese)	0.822	0.243	0.001^**^
Eggs	0.696	0.309	0.024^*^
Honey and other bee products	1.237	0.657	0.060
Value‐added products (jams)	0.077	0.360	0.831
Sustainable attributes			
Environmentally friendly	0.080	0.257	0.755
Local	0.237	0.350	0.499
Organic	0.608	0.302	0.044^*^
Method types			
Hypothetical	0.197	0.167	0.239
Cons	−2.922	0.724	0.000
Number of observations	47		
Tau^2^	0.052		
I^2^	99.53%		
H^2^	211.16		
R^2^	66.22		
Prob > F	0.0000*		

*** significance level = 0.001

** significance level = 0.01

* significance level = 0.05

Abbreviations: Confidence interval (CI); Tau‐squared (Tau^2^); I‐squared (I^2^); H‐squared (H^2^); R‐squared (R^2^); F‐statistics (Prob > F)

## 4 Discussion

In this study, we conducted a meta‐analysis to gather together structured data with regard to consumer WTP for SFSC products by reviewing and analyzing 47 articles. The analysis focused on the average WTP values for such products, to address the gaps in the meta‐analyses related to consumer WTP, and the literature on consumer behavior. Despite the data diversity, by compiling the work conducted to date, this study offers key insights that can guide future investigations on consumer behavior regarding SFSCs.

Overall, the results of the MRA indicated that gender, education, year of study, age group, region, product category, and sustainability attributes significantly influenced the average WTP estimates and contributed to the heterogeneity in consumer WTP. The overall calculated WTP was 34.5%, considered a medium estimate for SFSC products, and in line with the current price premiums for organic attributes worldwide (20%–40%).^[^ ^58^ ^]^ In the same way, Yang and Renwick^[^ ^67^ ^]^ conducted a study reporting a meta‐analysis WTP value of 36% for the organic attributes of livestock products, while Li and Kallas^[^ ^23^ ^]^ reported a WTP estimate of 29.5% for sustainable food products.

Our results highlight a notable and statistically significant effect of gender on the WTP estimates for products sourced from the SFSC sector. Specifically, the results indicate that women exerted a more significant influence on WTP estimates than men. This finding aligns with previous research which consistently demonstrates that women exhibit a stronger inclination toward pro‐environmental behaviors^[^ ^68^, ^69^ ^]^ and express higher levels of environmental concern compared to men.^[^ ^70^, ^71^ ^]^ Moreover, previous meta‐analyses have also indicated that women tend to exhibit a higher WTP for sustainable products when compared to men^[^ ^23^ ^]^ Similarly, studies by Rezai et al.^[^ ^72^ ^]^ and Laroche et al.^[^ ^73^ ^]^ have observed that women are more willing to pay a premium for green food and eco‐friendly products.

A significant positive relationship was noted between education levels and the WTP for products derived from SFSC, indicating that as education increases, consumers' WTP also increases. Numerous studies have explored the impact of education on consumer WTP for sustainable products. For instance, this finding is corroborated by Tianyu and Meng,^[^ ^74^ ^]^ who concluded that higher educational attainment significantly enhances both the incentive and amount of WTP for environmental enhancement, supporting the hypothesis that education plays a pivotal role in environmental protection efforts. Furthermore, Kotchen et al.^[^ ^75^ ^]^ discovered a significantly positive influence of education on WTP for policy measures addressing climate change across various policy instruments. They found that higher educational levels correlate with greater WTP for all considered policy instruments, underscoring education's critical role in garnering public support for climate change policies. Additionally, Graça et al.^[^ ^76^ ^]^ examined how educational factors, whether initiated by consumers or organizations, positively influence consumers’ willingness to buy green products and contribute to building a brand's green image.

The period before 2014 witnessed relatively low levels of consumer WTP for SFSC products; this may have been due to a worldwide economic crisis that impacted consumer behavior and preferences. The 2008 economic crisis harmed consumer WTP for environmental and sustainable claims, as evidenced by a decrease in WTP for climate change mitigation,^[^ ^77^ ^]^ a decline in green product purchases,^[^ ^78^ ^]^ and a reduction in support for environmental protection.^[^ ^79^ ^]^ The crisis also led to a decrease in WTP for environmental quality improvements.^[^ ^80^ ^]^ In terms of world GDP growth (annual %), there was steady economic growth beginning in 2014,^[^ ^81^, ^82^ ^]^ increasing consumer WTP. This suggests that economic issues can also affect consumer WTP. The concept of SFSCs was formally defined and regulated within the framework of the EU, particularly under its rural development policy for 2014–2020.^[^ ^5^, ^83^ ^]^ According to Jameson et al.^[^ ^84^ ^]^ the Boston Consulting Group's (BCG's) 2022 survey indicated that 82% of shipping customers were willing to pay a premium for zero‐carbon shipping. They also proposed that any future increase in consumer WTP should be catalyzed by regulation; this approach would not only continue to be a fundamental driver but also garner increasing importance. In addition, in 2015, 15% of the farms in the EU chose to sell over 50% of their produce directly to consumers.^[^ ^5^ ^]^ These study findings highlight the importance of regulations that can catalyze consumer WTP for SFSC products.

A positive coefficient is evident within the younger age group, suggesting that belonging to a younger age category correlates with an increased WTP for SFSC products. This implies that younger individuals may exhibit a greater propensity to spend money on SFSC items compared to their older counterparts. These results are consistent with previous research. For instance, Li and Kallas^[^ ^23^ ^]^ conducted a meta‐analysis based on 80 papers on consumer WTP for sustainable products, and observed a similar trend, with the younger generation displaying higher WTP while older respondents typically showed lower WTP. However, it is also important to note nuances within age groups. Darby et al.^[^ ^43^ ^]^ highlighted that older consumers often demonstrate a greater WTP across various attribute categories compared to younger consumers. Additionally, Vecchio^[^ ^85^ ^]^ and Laroche et al.^[^ ^73^ ^]^ found that older respondents showed a higher WTP for sustainable wines and environmentally friendly products.

Considering the significance of regional attributes in fostering SFSC development,^[^ ^11^ ^]^ it is noteworthy that consumers in both the EU and North America demonstrate significant WTP compared to other regions, indicating a stronger inclination toward the adoption and support of SFSCs within these regions. This observation is unsurprising given the prevalence of SFSCs in both the EU and North America. In the EU, SFSC development is gaining momentum, with a focus on local production and consumption to ensure fair remuneration for farmers. Additionally, the growing interest in local food systems, as evidenced by the rapid increase in the number of farmers’ markets since the 2000s, suggests the expanding influence of SFSCs in both the North American and the EU regions.^[^ ^86^ ^]^ Moreover, when considering European regions, it becomes apparent that Western Europe exhibits the highest inclination to pay more for SFSC products, as evidenced by the significant and relatively high coefficient compared to Southern and Northern Europe (Eastern Europe was excluded due to outliers). This outcome aligns with the results of WTP estimates, which consistently indicate that Western Europe demonstrates the highest WTP compared to other EU regions, alongside the abundance of studies conducted in the SFSC domain within this region. However, to fully grasp the nuances and implications of consumer behavior in SFSC markets across Europe, further research is warranted.

Our analysis revealed that products in specific food categories such as fruits and vegetables, meat and poultry, dairy products, and eggs, significantly influence consumers’ WTP. Several studies have underscored that SFSC products are more prevalent in categories such as fruits and vegetables, with consumers willing to pay more for these items due to factors such as their local origin, quality, and freshness.^[^ ^87^ ^]^ Similarly, Kneafsey et al.^[^ ^5^ ^]^ discovered that within SFSCs, fresh fruits and vegetables are the primary products involved, closely followed by dairy and animal products, as well as beverages. In the EU, a project investigating consumer perceptions and behavior regarding SFSCs indicated that consumers frequently purchased SFSC products, especially vegetables, fruits, eggs, honey, and bread.^[^ ^88^ ^]^

Lastly, the findings revealed that consumers exhibit a higher WTP for organic food than for products demonstrating other sustainable attributes, indicating a clear preference for organic products in the SFSC sector. This finding comes as no surprise, given the consistent evidence from various studies indicating that consumers are willing to pay a premium for organic products. For instance, Zander and Feucht^[^ ^89^ ^]^ highlighted that consumers had a greater WTP for organic production (14.8%) than for other sustainability attributes such as animal welfare (14%) and locality (12.6%). Similarly, based on an MRA of sustainable food products, Li and Kallas^[^ ^23^ ^]^ observed the highest WTP estimates for organic foods. Van Loo et al.^[^ ^90^ ^]^ also reported a notable trend, wherein consumer WTP was highest for organic food (27.0%), surpassing other attributes such as Rainforest Alliance certification (19.5%) and fair‐trade origins (15.8%). Additionally, Zander and Hamm^[^ ^91^ ^]^ and Nandi et al.^[^ ^92^ ^]^ both found that consumers were willing to pay higher prices for organic products with additional ethical attributes such as animal welfare and fair prices to farmers. This suggests that consumers prioritize organic products due to their perceived environmental and ethical benefits.

### 4.1 Theoretical and Practical Implications of the Study

The findings of this study have significant theoretical and practical implications for understanding consumer behavior and promoting sustainable food systems. From a theoretical perspective, our empirical insights contribute to consumer behavior theory by identifying key factors influencing consumer WTP for SFSC products, including gender, education, year of study, age, region, product type, and specific sustainability attributes. While supporting established theories on consumer behavior and sustainability, our findings also challenge existing frameworks by highlighting the need to incorporate a broader set of variables—such as regions and product categories—that are often overlooked in the literature. This comprehensive approach allows for a more nuanced understanding of WTP heterogeneity, suggesting that existing models should be refined to capture the diverse drivers of consumer preferences in SFSC contexts. Furthermore, traditional consumer behavior models often generalize the relationship between sustainability and WTP. Our findings reveal that different product categories evoke varying levels of consumer engagement with sustainability attributes, emphasizing the need for refined frameworks that account for these specific attributes. Additionally, the study highlights the importance of transparency and direct consumer‐producer relationships in enhancing trust, a critical factor in sustainable consumption patterns.

From a practical perspective, these findings provide actionable insights for businesses, marketers, policymakers, and consumer advocacy groups. Businesses and marketers can design targeted campaigns that highlight attributes such as “organic‐sourced ingredients”, “reduced carbon footprint”, and “direct support to local farmers” to align with consumer preferences. For example, regional marketing initiatives such as farmers’ markets featuring locally sourced meat and dairy, or online platforms promoting honey with superior sensory attributes, illustrate how such strategies can enhance consumer engagement. Furthermore, WTP studies provide valuable insights into the maximum price consumers are willing to pay for a product or service. This helps businesses set optimal prices that maximize revenue without deterring potential consumers. Insights from WTP studies can also guide product development by highlighting features that consumers value most. For example, businesses can prioritize the development of products with eco‐friendly packaging or enhanced sensory attributes based on consumer preferences. Additionally, WTP studies enable businesses to conduct competitive analysis, helping them understand how their pricing compares to that of competitors. This information is crucial for positioning products in the market and making strategic decisions about pricing adjustments. By aligning prices with consumer expectations, businesses can enhance consumer satisfaction and loyalty, and avoid overpricing, which can lead to dissatisfaction, or underpricing, which might reduce revenue. Policymakers can use these insights to create incentives for SFSC adoption such as tax benefits or grants for local producers and retailers. Supply chain actors can implement transparency tools such as blockchain technology for traceability or eco‐labels to meet consumer demand for sustainable products. These strategies not only enhance consumer trust but also improve supply chain visibility and producer profitability. Consumer advocacy groups can also leverage these findings to educate the public on the environmental, economic, and social benefits of supporting SFSCs. For instance, campaigns that link sustainable farming practices to biodiversity preservation or reduced carbon emissions can drive consumer awareness and support for SFSCs. These campaigners can also influence policy decisions by showcasing the broader societal benefits of SFSCs using evidence from this study.

### 4.2 Limitations and Future Research

The current study has a number of limitations which also present opportunities for future research. First, while our study specifically focused on analyzing consumer WTP for products sold within SFSCs, we acknowledge the value of including non‐SFSC products in future research. Doing so would provide a broader perspective on consumer preferences and behaviors across different supply chains. Future studies could also explore research questions such as: How do WTP and sustainability perceptions differ between SFSC and non‐SFSC products? What factors drive consumer preferences for one system over the other? Incorporating non‐SFSC studies would deepen our understanding of the relative value propositions of different types of supply chains and contribute to ongoing discussions on the role of alternative food systems in promoting sustainability and resilience in the agri‐food sector. Second, while our study addresses certain aspects of consumer heterogeneity, unexplored and unmeasured factors may have influenced the outcomes. Future research could consider variables such as household size, technology adoption (e.g., mobile apps or blockchain for improving transparency), and environmental awareness to determine their impact on purchasing decisions and the demand for locally sourced and sustainably produced foods. For example, future studies could examine how household size affects WTP for SFSC products and whether larger or smaller households prioritize sustainability differently. Similarly, research could explore how mobile apps connecting consumers with local farmers or blockchain technology influence trust and WTP for SFSC products. Additionally, most of the studies referenced in our analysis were conducted in EU countries, which might introduce geographical bias. Future research should include studies from underrepresented regions such as South America, Oceania, Asia, and Africa to capture a more comprehensive global understanding of consumer WTP for SFSC products and highlight regional differences in consumer behavior. Specific research questions could include: How do cultural and economic contexts influence WTP for SFSC products? Are there region‐specific factors that shape sustainability perceptions? To strengthen policies supporting sustainable food systems and gather more robust evidence, it is crucial to expand the scope and increase the number of relevant studies. As more studies emerge, we anticipate more in‐depth analyses of consumer heterogeneity in terms of WTP for SFSC products. Future research could use a combination of meta‐regression analyses and experimental designs, such as conjoint analysis, to further understand consumer trade‐offs between sustainability and product attributes. In conclusion, broadening the scope of future studies and refining methodological approaches will provide a clearer understanding of the factors influencing consumer WTP for SFSC products, ultimately contributing to the ongoing development of sustainable food systems globally.

## 5 Conclusions

In this study, we performed a comprehensive meta‐analysis of the existing literature to analyze consumers’ WTP for SFSC products. Our findings revealed that the average WTP premium stood at 34.5%, which is similar to premiums observed with regard to other sustainability claims in the food systems context. This aligns with the broader trends observed in studies focusing on sustainability‐related WTP, indicating a consistent pattern across various contexts. The economic inclination was influenced by several factors, primarily consumer demographics and product type. Fresh produce, meat and poultry, dairy products and eggs significantly influence WTP premiums. These insights presented as a result of our analysis have substantial implications for SFSC stakeholders, including producers, marketers, and policymakers. We recommend that producers of product categories such as fresh produce, meat and poultry, dairy products, and eggs consider engaging in direct sales to consumers. This direct interaction not only enhances consumer trust and loyalty but also allows producers to communicate the unique qualities and sustainability practices inherent to their products. Moreover, participating in farmers’ markets, selling directly on farms, and utilizing other direct‐to‐consumer channels can enhance visibility and interaction between farmers and consumers. Such an approach can improve farmers’ profit margins and strengthen their relationship with their customer base while ensuring that consumers receive fresh and high‐quality locally‐sourced produce.

However, our study has several limitations that should be acknowledged. First, most of the studies included in our analysis were conducted in high‐income regions, which may limit the generalizability of our findings to lower‐income regions or different cultural contexts. Future research should aim to include a more geographically diverse sample to capture a broader range of consumer behavior across different economic contexts. Additionally, while our meta‐analysis focused on peer‐reviewed publications to ensure the highest level of credibility, this may have resulted in the exclusion of valuable insights from the grey literature or industry reports. Expanding future studies to include such sources could provide a more comprehensive understanding of consumer WTP for SFSC products. Finally, while we identified key demographic and product‐related factors influencing WTP, further research is needed to examine how household size, technology adoption, environmental awareness, and consumers’ understanding of sustainability shape preferences. Exploring these factors could enhance the understanding of the drivers behind consumer behavior, offering more targeted and effective strategies for SFSC stakeholders. On a global scale, the demand for such products is strongly connected with the cultural identity, food security, and sustainability goals of numerous nations. Looking to the future, our study highlights the need for further research into the efficiency and scalability of SFSCs within different economic and cultural contexts. Including non‐SFSC products in future studies would provide valuable insight into consumer preferences across supply chains, offering a comparative perspective. In addition, our analysis of methodological approaches reveals a critical area for future research as a means of refining WTP estimation techniques, ensuring that they are robust and free from bias.

In conclusion, this study not only provides actionable insights for SFSC stakeholders but also lays the groundwork for future research that could shape the development of local, national, and global food systems. By addressing the limitations and embracing a forward‐looking approach, this study contributes to enriching the literature on SFSCs and consumer behavior.

## Conflict of Interest

The authors declare no conflict of interest.

## Supporting information



Supplemental Table 1

## Data Availability

The data that support the findings of this study are available from the corresponding author upon reasonable request.
